# Exometabolite Dynamics over Stationary Phase Reveal Strain-Specific Responses

**DOI:** 10.1128/mSystems.00493-20

**Published:** 2020-12-22

**Authors:** John L. Chodkowski, Ashley Shade

**Affiliations:** a Department of Microbiology and Molecular Genetics, Michigan State University, East Lansing, Michigan, USA; b Department of Plant, Soil and Microbial Sciences, Michigan State University, East Lansing, Michigan, USA; c Program in Ecology, Evolution, and Behavior, Michigan State University, East Lansing, Michigan, USA; Institute for Systems Biology

**Keywords:** *Burkholderia thailandensis*, *Chromobacterium violaceum*, *Pseudomonas syringae*, secondary metabolism, RNA-seq, mass spectrometry, metabolomics, stationary phase, nongrowth state, persistence

## Abstract

Microbial exponential growth is expected to occur infrequently in environments that have long periods of nutrient starvation punctuated by short periods of high nutrient flux. These conditions likely impose nongrowth states for microbes. However, nongrowth states are uncharacterized for the majority of environmental bacteria, especially in regard to exometabolite production. We compared exometabolites produced over stationary phase across three environmental bacteria: Burkholderia thailandensis E264 (ATCC 700388), Chromobacterium violaceum ATCC 31532, and Pseudomonas syringae pv. *tomato* DC3000 (ATCC BAA-871). We grew each strain in monoculture and investigated exometabolite dynamics from mid-exponential to stationary phases. We focused on exometabolites that were released into the medium and accumulated over 45 h, including approximately 20 h of stationary phase. We also analyzed transcripts (transcriptome sequencing [RNA-seq]) to interpret exometabolite output. We found that the majority of exometabolites released were strain specific, with a subset of identified exometabolites involved in both central and secondary metabolism. Transcript analysis supported that exometabolites were released from intact cells, as various transporters had either increased or consistent transcripts through time. Interestingly, we found that succinate was one of the most abundant identifiable exometabolites for all strains and that each strain rerouted their metabolic pathways involved in succinate production during stationary phase. These results show that nongrowth states can be metabolically dynamic and that environmental bacteria can enrich a minimal environment with diverse chemical compounds as a consequence of growth and postgrowth maintenance in stationary phase. This work provides insights into microbial community interactions via exometabolites under conditions of growth cessation or limitation.

**IMPORTANCE** Nongrowth states are common for bacteria that live in environments that are densely populated and predominantly nutrient exhausted, and yet these states remain largely uncharacterized in cellular metabolism and metabolite output. Here, we investigated and compared stationary-phase exometabolites and RNA transcripts for each of three environmental bacterial strains. We observed that diverse exometabolites were produced and provide evidence that these exometabolites accumulate over time through release by intact cells. Additionally, each bacterial strain had a characteristic exometabolite profile and exhibited dynamics in exometabolite composition. This work affirms that stationary phase is metabolically dynamic, with each strain tested creating a unique chemical signature in the extracellular space and altering metabolism in stationary phase. These findings set the stage for understanding how bacterial populations can support surrounding neighbors in environments with prolonged nutrient exhaustion through exometabolite-mediated interspecies interactions.

## Abstract (precis)

Nongrowth states are common for bacteria that live in environments that are densely populated and predominantly nutrient exhausted, and yet these states remain largely uncharacterized in cellular metabolism and metabolite output. Here, we investigated and compared stationary-phase exometabolites and RNA transcripts for each of three environmental bacterial strains.

## INTRODUCTION

Much of microbiology research in the laboratory is conducted with bacterial or archaeal populations that are growing exponentially. However, it is estimated that 60% of the microbial biomass in the environment is in a nongrowing state (1, 2). Nongrowing states can arise by virtue of being dormant (e.g., low metabolic activity) or entering stationary phase (e.g., maintenance levels of metabolic activity) (3), where the latter refers to a population-level phenomenon that occurs after exponential growth. Various abiotic and biotic stressors at carrying capacity are known to induce stationary phase, including nutrient exhaustion/inaccessibility and the accumulation of waste products. Particular environments impose conditions where microbial populations are in stationary phase for a better part of their existence, for example, dry soils with intermittent periods of rewetting (4–6), activated sludge operating in a sequencing batch reactor (SBR) (7, 8), and the human gut (9, 10). Thus, unlike most cultivated laboratory strains, microbes experience stationary phase in environments where short periods of high nutrient flux are followed by long periods of famine (11, 12).

Bacteria survive in stationary phase by employing various stress response adaptations (13–15). Stress response adaptations include changes to cell morphology, transcription, translation, and metabolism. Furthermore, in stationary phase, microbes can reroute metabolic pathways to maintain essential components of the cell and the proton motive force (PMF) (16). While these adaptations are thought to serve as survival mechanisms, the levels and types of metabolic activities in stationary phase are not well understood for most environmental microbes.

It is known, however, that microbes can exhibit appreciable metabolic activity in stationary phase (17). For example, entry into stationary phase resulted in prolonged protein production in Escherichia coli despite a decrease in overall protein levels (18). Metabolomic studies of E. coli in stationary phase support that there are unique metabolite production profiles associated with metabolic responses to growth arrest (19–21). These studies have provided valuable insights into stationary-phase physiology. However, metabolome studies of microorganisms have generally focused on the dynamics of intracellular metabolites. It is expected that understanding metabolite dynamics in the extracellular environment can provide insights into metabolic responses that are relevant for microbial communities and interactions among coexisting community members.

Exometabolomics is the characterization of small, extracellular molecules released by a microbe by means of either lysis or diffusion, passive or active (22). Characterizing exometabolites can provide insights into the potential for microbes to engage locally with other microbes and the environment via the release of small molecules (23). The effect of these small molecules on neighboring microbes can range from cooperative (e.g., signaling molecules) to antagonistic (e.g., antibiotics) (24). Some exometabolites, such as antibiotics, are known to increase in production upon entry into stationary phase (15). In addition, computational models have predicted that costless exometabolite production, such as central carbon metabolism, may be common among bacteria (25), which could provide an overall benefit in a microbial community setting. Untargeted exometabolomic profiling has benefited from recent advances in the sensitivity and throughput of mass spectrometers (26). This approach provides an experimental basis to observe the breadth of exometabolites produced by microbial strains and strain-specific contributions to the exometabolite pool. Characterizing the exometabolite profile of a microbial population over time can be applied to understand the dynamic interplay between cell metabolism and the environment. Integrating untargeted exometabolomic approaches with other omics technologies (e.g., transcriptomics and genomics) informs comparisons across microbial populations of their metabolic responses in stationary phase.

We present an investigation of three environmental bacterial strains that are commonly associated with terrestrial environments (soils or plants) (Table 1). These strains were chosen because of reported (27) and observed interspecies exometabolite interactions in the laboratory. This study evaluated exometabolite production for each strain in monoculture to first establish typical single-strain responses over stationary phase, with goals to next proceed to understand exometabolite-mediated interactions among strains. Our previous work established a robust and flexible approach to investigate microbial exometabolite production in either monoculture or coculture (28). Our approach uses filter plates that allow the separation of cells from an exometabolite reservoir. Here, we examined the detailed exometabolite and transcript dynamics, defined as compositional changes through time, for each of these three environmental strains in monoculture over stationary phase after growth in minimal glucose (3.7 mM) medium. We asked the following questions. What is the diversity of unique exometabolites that accumulate over stationary phase? What is a likely explanation (e.g., transport from viable cells or lysis) for the accumulation of exometabolites? How do exometabolite composition and production compare across strains and time, and what general insights could these provide for understanding microbial metabolism and ecology in stationary phase?

**TABLE 1:** Bacterial strains used in this study

Strain (reference)	Family	Genome size (Mb)	No. of ORFs^*a*^
Burkholderia thailandensis E264 (68)	*Burkholderiaceae*	6.72	5,641
Chromobacterium violaceum ATCC 31532 (69)	*Neisseriaceae*	4.75	4,371
Pseudomonas syringae pv. *tomato* DC3000 (70)	*Pseudomonadaceae*	6.53	5,853

a ORFs, open reading frames.

We found that exometabolite composition is dynamic through stationary phase and that accumulated exometabolites were likely released from intact cells. We also found that a majority of released exometabolites were strain specific, suggesting that different bacterial strains have individualized responses to stationary phase. Finally, we found that all three strains rerouted metabolism in stationary phase.

## RESULTS

### Each strain had a distinct exometabolite profile in stationary phase.

**FIG 1:**
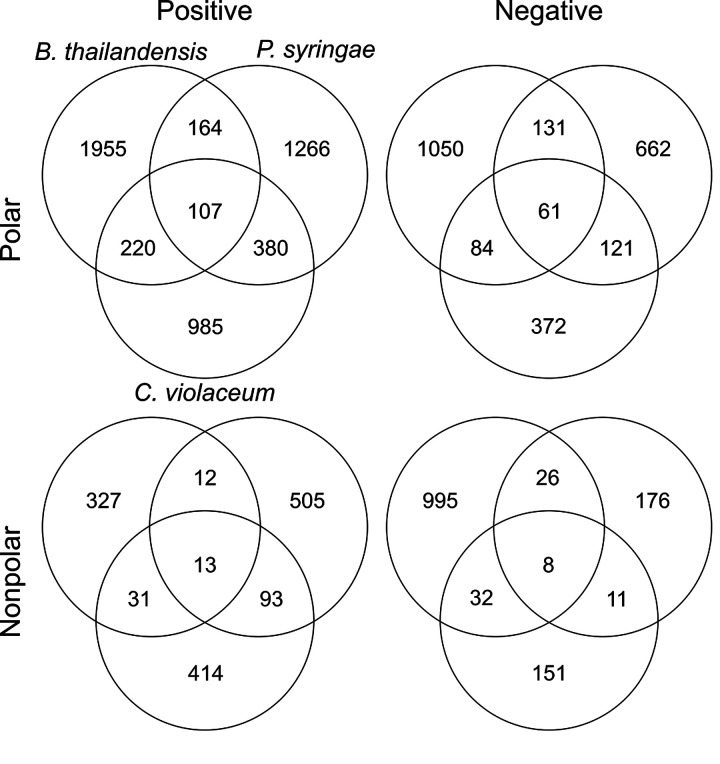
Quantification of all features that fit criteria for released in all strains across all polarity/ionization modes.

In total, 10,352 features were detected by mass spectral analysis (Fig. 1 and Table 2) across the three strains. These features represent what we defined as released exometabolites (see the mass spectrometry analysis section in Materials and Methods). Briefly, released exometabolites were defined as those that had temporal accumulation (assessed via peak area) in stationary phase. Most features detected were strain specific, and the number of unique features from any one strain outnumbered the total number of features shared by at least two strains (1,494 features; ∼16.9%). Of the 1,494 shared features, ∼12.7% were shared among all three strains. Specifically, Burkholderia thailandensis had the most unique detected features (∼41.8%), followed by Pseudomonas syringae (∼25.2%) and Chromobacterium violaceum (∼18.6%), compared to all detected features. These data suggest that despite monoculture growth in minimal medium initially containing one carbon source, an abundance of strain-specific exometabolites are produced during stationary phase.

**TABLE 2:** Summary of released exometabolites for each strain

Parameter	Value for organism		
	B. thailandensis	C. violaceum	P. syringae
Total no. of features	5,216	3,083	3,736
No. of unique features	4,327	1,922	2,609
No. of features in common with B. thailandensis		367	333
No. of features in common with C. violaceum	367		605
No. of features in common with P. syringae	333	605	
No. of features detected in all strains	189	189	189

**FIG 2:**
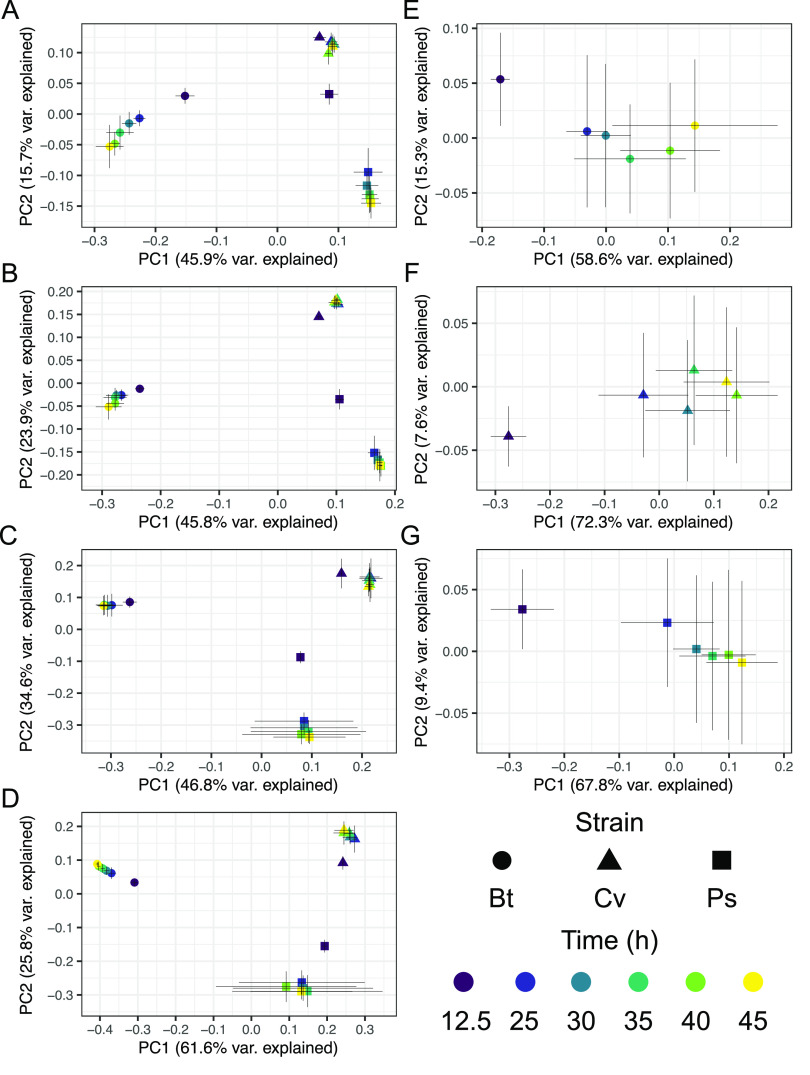
Exometabolite profiles differ by strain and time. Shown are PCoA plots for polar positive (A), polar negative (B), nonpolar positive (C), nonpolar negative (D), and combined polar positive and polar negative exometabolites (accounting for 72 to 77% of the released exometabolites per strain) for B. thailandensis (Bt) (E), C. violaceum (Cv) (F), and P. syringae (Ps) (G). Each point represents the exometabolite profile (relative contributions assessed by peak area) for a particular strain at a particular time point. Features were normalized by an internal standard (ITSD) reference feature and cube root transformed. The Bray-Curtis distance metric was used to calculate dissimilarities between exometabolite profiles. Strain is indicated by shape, and time point is indicated by a color gradient. Error bars are 1 standard deviation around the mean axis scores from 2 to 4 replicates destructively sampled from the same strain/time point conditions.

We were interested in understanding differences in exometabolite composition and exometabolite temporal dynamics over stationary phase (Fig. 2). Comparing across strains (Fig. 2A to D), each strain had strain-specific exometabolite profiles (0.590 ≤ *r*^2^ ≤ 0.808 by Adonis; *P* value, ≤0.001; all pairwise false discovery rate [FDR]-adjusted *P* values, ≤0.001). For each strain, exometabolite profiles from the exponential growth phase were distinct from the stationary-phase profiles (Fig. 2). Strain differences in released exometabolites were more important than time in explaining the variation in exometabolite composition on both principal-coordinate analysis (PCoA) axes. As expected, strain identity explained ≥57% of the variation, while time explained ≤6% of the variation, across all polarity/ionization modes (see Table S1 in the supplemental material). However, the most variation was explained by the interaction effect of strain × time (Table S1). Thus, exometabolite compositional differences were mainly driven by the different exometabolites released by the different strains. This was expected given the large number of unique features detected for each strain (Table 2).

10.1128/mSystems.00493-20.5 TABLE S1 Percent variation explained on the effects of strain, time, and their interaction on exometabolite profiles. Permutational multivariate analysis of variance (PERMANOVA) revealed strain-specific differences in exometabolite composition (all *P ≤ *0.001). Download Table S1, DOCX file, 0.02 MB. Copyright © 2020 Chodkowski and Shade. 2020 Chodkowski and Shade. This content is distributed under the terms of the Creative Commons Attribution 4.0 International license.

Alternatively, we further looked at the influence of time on exometabolite profiles by observing exometabolites released by each strain, separately. We considered only those exometabolites that met our stringent criteria for release and accumulation over time (see Materials and Methods). Notably, with these criteria, some of the same exometabolites were classified as released for some strains but not for others. In these cases, exometabolites were excluded from the temporal analysis of any strains for which the release criteria were not met. Directional temporal dynamics were observed for each strain (Fig. 2E to G), although continued directionality was not observed at some of the latest time points (e.g., Fig. 2F). We define directional as a progressive, stepwise trajectory between time points where each time point is distinguished from any of the previous time points and even more distinct from previous time points in PCoA space. This ultimately reflects temporal changes in exometabolite composition. Temporal trajectories in exometabolite profiles were highly reproducible for each strain across biological replicates (Protest analyses) (Table S2). For all strains, the difference between exometabolite profiles progressively increased when each stationary-phase time point was compared to the initial, exponential-phase time point (Table S3). But comparing successive time points revealed that the greatest differences occurred between the first stationary-phase time point and the exponential-phase time point. Notably, dissimilarity decreased between successive time points in stationary phase such that the latest time points were more similar to each other than the earliest time points (Table S4). For each strain, the exometabolite profile changed over time (Table S5). However, this was primarily due to differences in exometabolite profiles when the exponential-phase time point was compared to each of the stationary-phase time points (Table S6). We note that hundreds to thousands of features were detected in late stationary phase but were excluded (see the mass spectrometry analysis section in Materials and Methods) from the final data set of released exometabolites. We maintained strict criteria for the detection and accumulation of released exometabolites over stationary phase. Taken together, these data suggest that differences in exometabolite composition are largely driven by strain-specific production of exometabolites. Accounting for all released exometabolites within each strain, similar temporal patterns emerge, with the largest differences being observed between exponential phase and stationary phase and more subtle differences being observed over consecutive time points within stationary phase.

10.1128/mSystems.00493-20.6 TABLE S2 Summary of Protest analyses comparing exometabolite compositions through time across independent replicates. Coordinates of the first two PCoA axes were used to perform Protest analyses. Ranges reflect separate Protest analyses performed for each polarity (polar/nonpolar) and ionization mode (positive/negative). Download Table S2, DOCX file, 0.02 MB. Copyright © 2020 Chodkowski and Shade. 2020 Chodkowski and Shade. This content is distributed under the terms of the Creative Commons Attribution 4.0 International license.

10.1128/mSystems.00493-20.7 TABLE S3 Average Bray-Curtis dissimilarity between group centroids comparing each stationary-phase time point to the initial, exponential-phase time point (12.5 h). Ranges reflect separate analyses performed for each polarity (polar/nonpolar) and ionization mode (positive/negative). Download Table S3, DOCX file, 0.02 MB. Copyright © 2020 Chodkowski and Shade. 2020 Chodkowski and Shade. This content is distributed under the terms of the Creative Commons Attribution 4.0 International license.

10.1128/mSystems.00493-20.8 TABLE S4 Average Bray-Curtis dissimilarity between group centroids comparing time points in a stepwise manner. Ranges reflect separate analyses performed for each polarity (polar/nonpolar) and ionization mode (positive/negative). Download Table S4, DOCX file, 0.02 MB. Copyright © 2020 Chodkowski and Shade. 2020 Chodkowski and Shade. This content is distributed under the terms of the Creative Commons Attribution 4.0 International license.

10.1128/mSystems.00493-20.9 TABLE S5 Repeated-measures PERMANOVA performed on independently replicated time series within each strain. *P* values are listed, followed by *R*^2^ values in parentheses. Download Table S5, DOCX file, 0.02 MB. Copyright © 2020 Chodkowski and Shade. 2020 Chodkowski and Shade. This content is distributed under the terms of the Creative Commons Attribution 4.0 International license.

10.1128/mSystems.00493-20.10 TABLE S6 *Q* values from pairwise Adonis tests comparing all time points within a strain. Ranges reflect separate analyses performed for each polarity (polar/nonpolar) and ionization mode (positive/negative). Download Table S6, DOCX file, 0.02 MB. Copyright © 2020 Chodkowski and Shade. 2020 Chodkowski and Shade. This content is distributed under the terms of the Creative Commons Attribution 4.0 International license.

**FIG 3:**
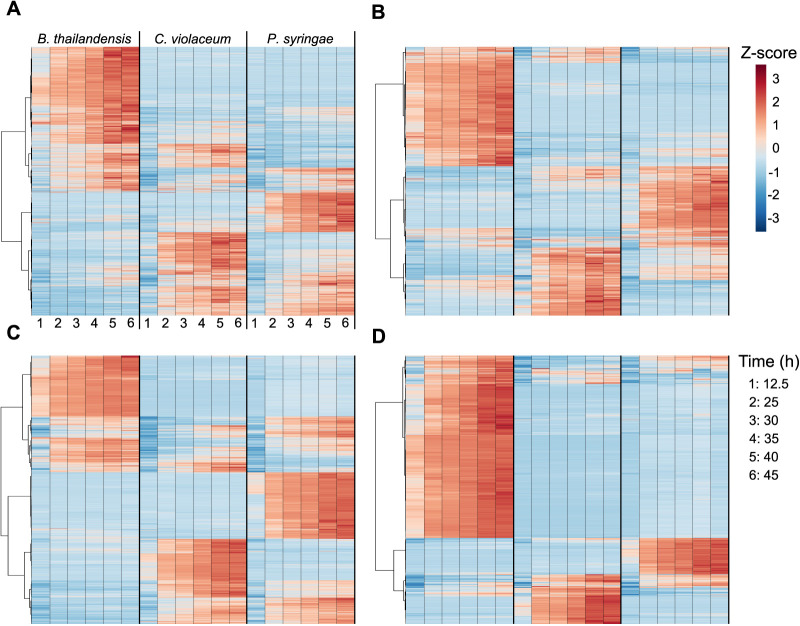
Released exometabolites and their temporal dynamics. A heat map of all released exometabolites is shown for polar positive (A), polar negative (B), nonpolar positive (C), and nonpolar negative (D) modes, where samples are in columns and exometabolites are in rows. Data for each sample are the averages from independent time point replicates (*n* = 2 to 4). Euclidean distance was calculated from Z-scored mass spectral profiles (containing peak areas). Prior to Z-scoring, features were normalized by an ITSD reference feature and cube root transformed. Features were clustered by Ward’s method.

Hierarchal clustering analysis also revealed strain-specific features and their dynamics (Fig. 3). Most features across all strains reached maximum accumulation in late stationary phase. Notably, exometabolites accumulated despite generally steady strain population levels (Fig. S1). We observed ∼1 generation in B. thailandensis and P. syringae over the course of stationary phase, but the doubling took 20 h to complete. Dead cells across the time series remained consistent for both B. thailandensis and C. violaceum but increased for P. syringae (Fig. S1). However, the quantity of live cells remained higher than the quantity of dead cells across the time series for all strains. The largely consistent counts of viable cells and the lack of a death phase suggest that many exometabolites were released by intact cells rather than by lysis. To add support to this hypothesis, transcriptomics data indicate that multiple organic molecule transporters were either consistently expressed throughout the time series or differentially expressed (Table 3; see also Dataset 1 at https://github.com/ShadeLab/Paper_Chodkowski_MonocultureExometabolites_2020/tree/master/Datasets). Notable examples for all strains include various transporters related to dipeptide and C-4 dicarboxylate transport. In summary, despite growth arrest, each bacterial strain continued to produce (and the media accumulated) a distinctive and dynamic profile of exometabolites into stationary phase.

**TABLE 3:** Summary of RNA-seq results with a focus on genes annotated as transporters^*d*^

Gene category	No. of genes								
	B. thailandensis			C. violaceum			P. syringae		
Genes involved in transport	669			465			689		
	447^*a*^	103^*a*^^,^^*b*^	20^*a*^^,^^*b*^^,^^*c*^	354^*a*^	169^*a*^^,^^*b*^	53^*a*^^,^^*b*^^,^^*c*^	461^*a*^	136^*a*^^,^^*b*^	12^*a*^^,^^*b*^^,^^*c*^

Genes annotated as transporters related to dipeptide/C-4 dicarboxylate transport	26			22			43		
	17^*a*^	4^*a*^^,^^*b*^	0^*a*^^,^^*b*^^,^^*c*^	22^*a*^	7^*a*^^,^^*b*^	1^*a*^^,^^*b*^^,^^*c*^	20^*a*^	10^*a*^^,^^*b*^	0^*a*^^,^^*b*^^,^^*c*^

Genes annotated as transporters related to dipeptide/C-4 dicarboxylate transport (transcripts below LEM)	9			0			23		

a Above the low-expression minimum (LEM).

b Differentially expressed (*Q* value of <0.01).

c Log_2_ fold change (LFC) of >1.

d Criteria included (i) genes that were above the low-expression minimum, (ii) genes that were differentially expressed, and (iii) genes with a stationary-phase time point that had an LFC of >1 compared to the exponential-phase time point.

10.1128/mSystems.00493-20.1 FIG S1 Counts of live (green) and dead (blue) cells throughout the time course. Cells were obtained from 5 wells in the transwell plate for 5 technical replicates/independent replicate at each time point. Syto9- and propidium iodide-stained cells were counted using flow cytometry. Download FIG S1, EPS file, 0.03 MB. Copyright © 2020 Chodkowski and Shade. 2020 Chodkowski and Shade. This content is distributed under the terms of the Creative Commons Attribution 4.0 International license.

### Identity of stationary-phase exometabolites.

**FIG 4:**
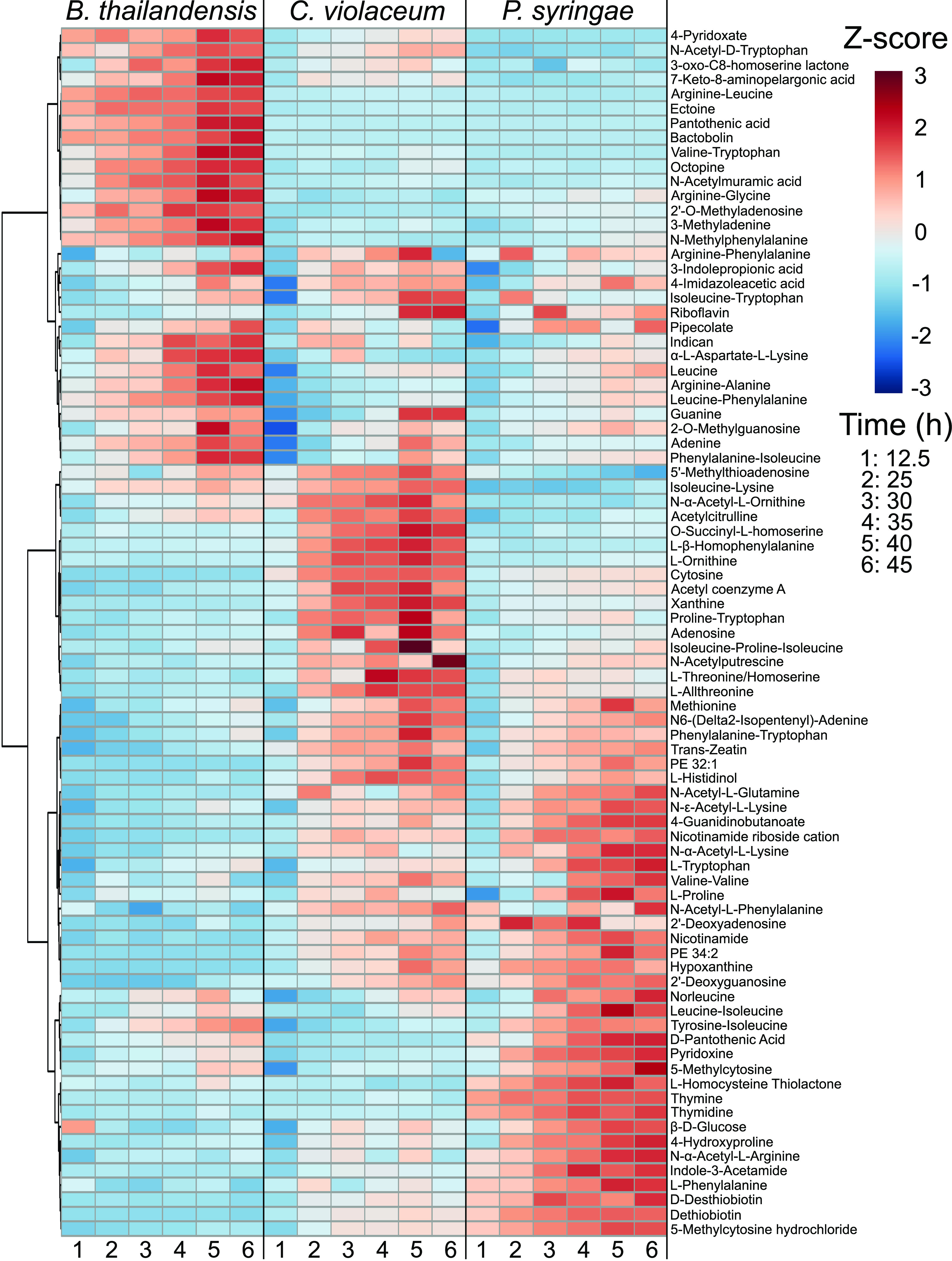
Released and identified exometabolites and their temporal dynamics. A heat map of identified exometabolites in polar positive mode is shown, where samples are in columns and exometabolites are in rows. Data for each sample are the averages from independent time point replicates (*n* = 3 or 4). Euclidean distance was calculated from Z-scored mass spectral profiles (containing peak areas). Prior to Z-scoring, features were normalized by an ITSD reference feature and cube root transformed. Features were clustered by Ward’s method.

Of the total set of exometabolite features, only 188 (∼1.8%) could be identified (Fig. 4 and Fig. S2 to S4; see also Dataset 2 at https://github.com/ShadeLab/Paper_Chodkowski_MonocultureExometabolites_2020/tree/master/Datasets). These were classified according to the Metabolomics Standards Initiative (MSI): MSI level 1 (identified compounds) and MSI level 2 (putatively identified compounds). Most of the identified exometabolites were uniquely produced by one strain under our experimental conditions, although there were some exometabolites that were shared across strains, especially between C. violaceum and P. syringae (see Dataset 2 at the URL mentioned above). Many of the identified exometabolites, particularly those molecules involved in central metabolism, such as amino acids, nucleotides/nucleosides, and carboxylic acids, were classified using an in-house standard in accordance with MSI level 1. In addition, MSI level 1 exometabolites such as ectoine, proline, trehalose, and glutamate likely indicated cellular stress (e.g., osmotic stress).

10.1128/mSystems.00493-20.2 FIG S2 Released and identified exometabolites and their temporal dynamics. A heat map of identified exometabolites in polar negative mode is shown, where samples are in columns and exometabolites are in rows. Results for each sample are the averages from independent time point replicates (*n* = 3 or 4). Euclidean distance was calculated from Z-scored mass spectral profiles (containing peak areas). Prior to Z-scoring, features were normalized by an internal standard (ITSD) reference feature and cube root transformed. Features were clustered by Ward’s method. Download FIG S2, EPS file, 3.5 MB. Copyright © 2020 Chodkowski and Shade. 2020 Chodkowski and Shade. This content is distributed under the terms of the Creative Commons Attribution 4.0 International license.

Exometabolites putatively identified at MSI level 2 were annotated by matching tandem mass spectrometry (MS/MS) fragmentations to a reference database. MSI level 2 exometabolites included secondary metabolites such as bactobolin, yersiniabactin, and acyl homoserine lactones (AHLs) produced by B. thailandensis, P. syringae, and C. violaceum, respectively. Bactobolin and yersiniabactin are bioactive molecules previously characterized as a bacteriostatic antibiotic (29) and a siderophore/virulence factor (30), respectively. AHLs induce quorum sensing in C. violaceum and are linked to the production of hydrogen cyanide, antibiotics, and proteases (31, 32). These putatively identified secondary exometabolites suggest that stationary phase is coordinated with shifts in metabolism, priming strains for competition via chemical warfare or nutrient scavenging. These data also suggest that a competitive phenotype may be standard among bacteria even in the absence of non-kin competitors, suggesting either priming for interspecific competition or engagement in intraspecific competition. This competitive priming is also supported by the observation of increased transcripts for transport systems involved in competition. For example, competitive transport systems included the type III secretion system in B. thailandensis and multidrug efflux systems for both C. violaceum and P. syringae. When transcripts between times of 45 h and 12.5 h were compared, the above-mentioned transport systems had a log_2_ fold change (LFC) in expression of >1 (see Dataset 1 at https://github.com/ShadeLab/Paper_Chodkowski_MonocultureExometabolites_2020/tree/master/Datasets). Finally, a large proportion of MSI level 2 exometabolites were dipeptides, suggesting either the degradation of proteins (14) or the formation of dipeptides by nonribosomal peptide synthetases (NRPSs), found in biosynthetic gene clusters (BSGCs) (see Dataset 3 at the URL mentioned above). In summary, there was a consistent accumulation of a diversity of exometabolites in stationary phase, including exometabolites that were intermediates in central carbon metabolism as well as secondary metabolites implicated in competition.

**FIG 5:**
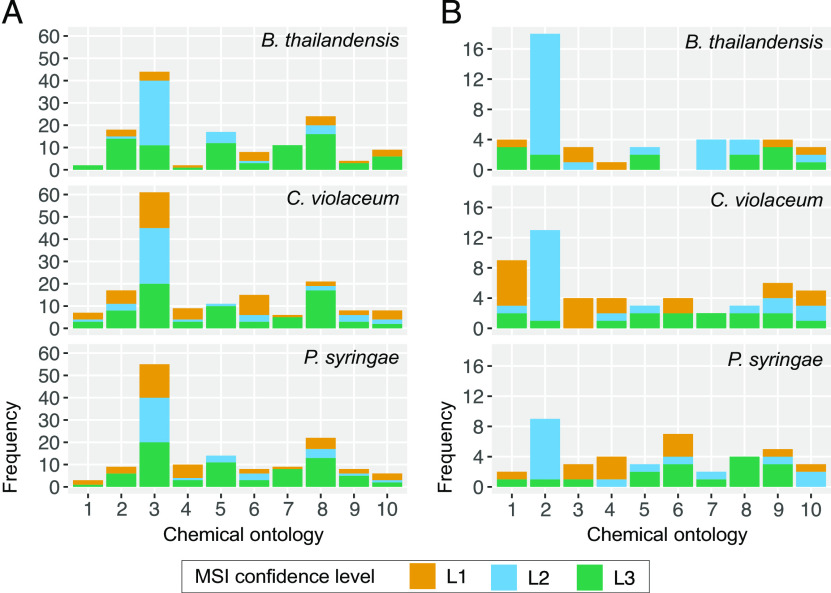
Chemical ontologies at different MSI levels. ClassyFire was used to categorize identified (MSI level 1 and level 2) and *in silico*-predicted (MSI level 3) MS/MS data at the class (A) and direct parent (B) levels. Identification confidence levels 1, 2, and 3 refer to Metabolomics Standards Initiative (MSI) identification levels 1, 2, and 3, respectively. The top 10 chemical ontologies are provided for each classification level. Chemical ontologies for panel A: 1, azoles; 2, benzene and substituted derivatives; 3, carboxylic acids and derivatives; 4, diazines; 5, fatty acyls; 6, imidazopyrimidines; 7, organonitrogen compounds; 8, organooxygen compounds; 9, purine nucleosides; 10, pyridines and derivatives. Chemical ontologies for panel B: 1, alpha amino acids; 2, dipeptides; 3, hydroxybenzoic acid derivatives; 4, hydroxypyrimidines; 5, medium-chain fatty acids; 6, *N*-acyl-alpha amino acids; 7, *N*-acyl-alpha amino acids and derivatives; 8, peptides; 9, purine nucleosides; 10, 6-alkylaminopurines.

To maximize the annotation of the remaining unidentified MS/MS data, we performed chemical ontology analysis to determine chemical classes of exometabolites produced in stationary phase. Using *in silico* prediction of exometabolites by MS/MS fragmentation patterns, we putatively characterized compound classes (MSI level 3 designation). Broadly, carboxylic acids and derivatives were the most abundant type of exometabolite produced in stationary phase for all strains (Fig. 5A). This is expected because carboxylic acid derivatives are prominent in cellular constituents and molecules involved in primary metabolism (e.g., tricarboxylic acid [TCA] cycle). Their excess production, and release to relieve internal accumulation, may be due to stoichiometric constraints in metabolic network topology (33). However, MSI level 3 exometabolites revealed a considerable quantification of exometabolites related to fatty acyls, organonitrogen compounds, organooxygen compounds, and benzene and substituted derivatives, suggesting additional classes of exometabolites contributing to the exometabolite pool that are unable to be identified by MSI level 1 and level 2 standards. These chemical ontologies were resolved further to the direct parent level (Fig. 5B). Amino acids and peptides were the most abundant and common exometabolites across all identification levels. In particular, dipeptides were the most abundant exometabolites. Transcriptomics data also indicated that dipeptide transporters for each strain were either consistently expressed or differentially expressed over time (Table 3; see also Dataset 1 at the URL mentioned above). In summary, chemical ontology analysis revealed chemical classes represented in the exometabolite data set but lacking identification and revealed that dipeptides were a common exometabolite released by all strains.

### Insights into stationary-phase metabolic rerouting.

**FIG 6:**
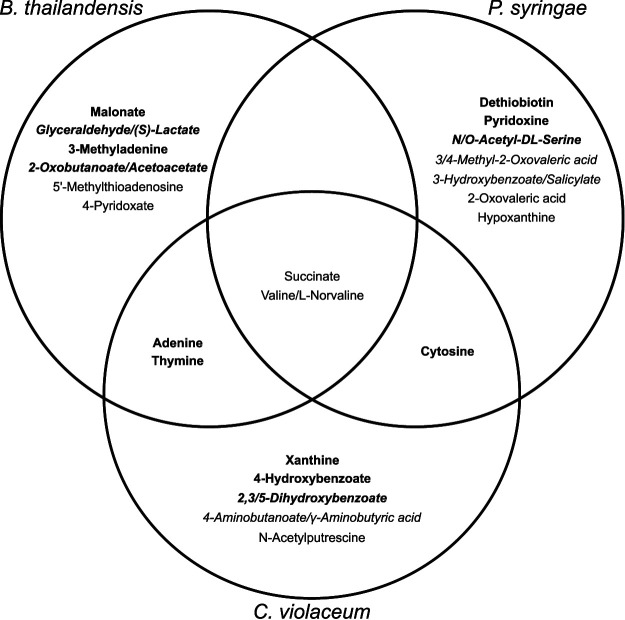
Distinctions and overlaps between the most abundant exometabolites in each strain. Exometabolites in boldface type passed the criteria for released. Exometabolites in italic type are isomers and could not be resolved to determine the exact identification.

We then aimed to interpret strain metabolism in stationary phase by focusing on the exometabolites most confidently identified (MSI level 1). For each strain, we examined the 10 most abundant exometabolites that accumulated and were detected at the last time point (45 h) for positive and negative polar exometabolites. We included all MSI level 1 exometabolites in this analysis. Generally, the abundant, accumulated exometabolites that were distinct for each strain were also uniquely detected in those strains (Fig. 6) (all *Q* values were ≤0.01 by analysis of variance [ANOVA]), with two exceptions: 5′-methylthioadenosine and hypoxanthine were also abundant in Chromobacterium violaceum media but not within its top 10 accumulated exometabolites. Transporters that had LFCs in expression (comparing times of 45 h and 12.5 h) of >1 could be linked with their substrates for both C. violaceum and P. syringae (see Dataset 1 at https://github.com/ShadeLab/Paper_Chodkowski_MonocultureExometabolites_2020/tree/master/Datasets). This included substrates such as succinate and cytosine for C. violaceum and P. syringae, respectively. None of the most abundant exometabolites in B. thailandensis could be linked to a transporter with a high LFC. The majority of strain-specific abundant exometabolites suggested that each strain released a set of unique metabolic intermediates into the extracellular environment. This finding could have implications for how bacterial populations maintain viability through interspecies interactions in periods of nutrient exhaustion. Perhaps a simple explanation for differences in the types of exometabolites released could be differences in the alteration of stationary-phase metabolism.

**FIG 7:**
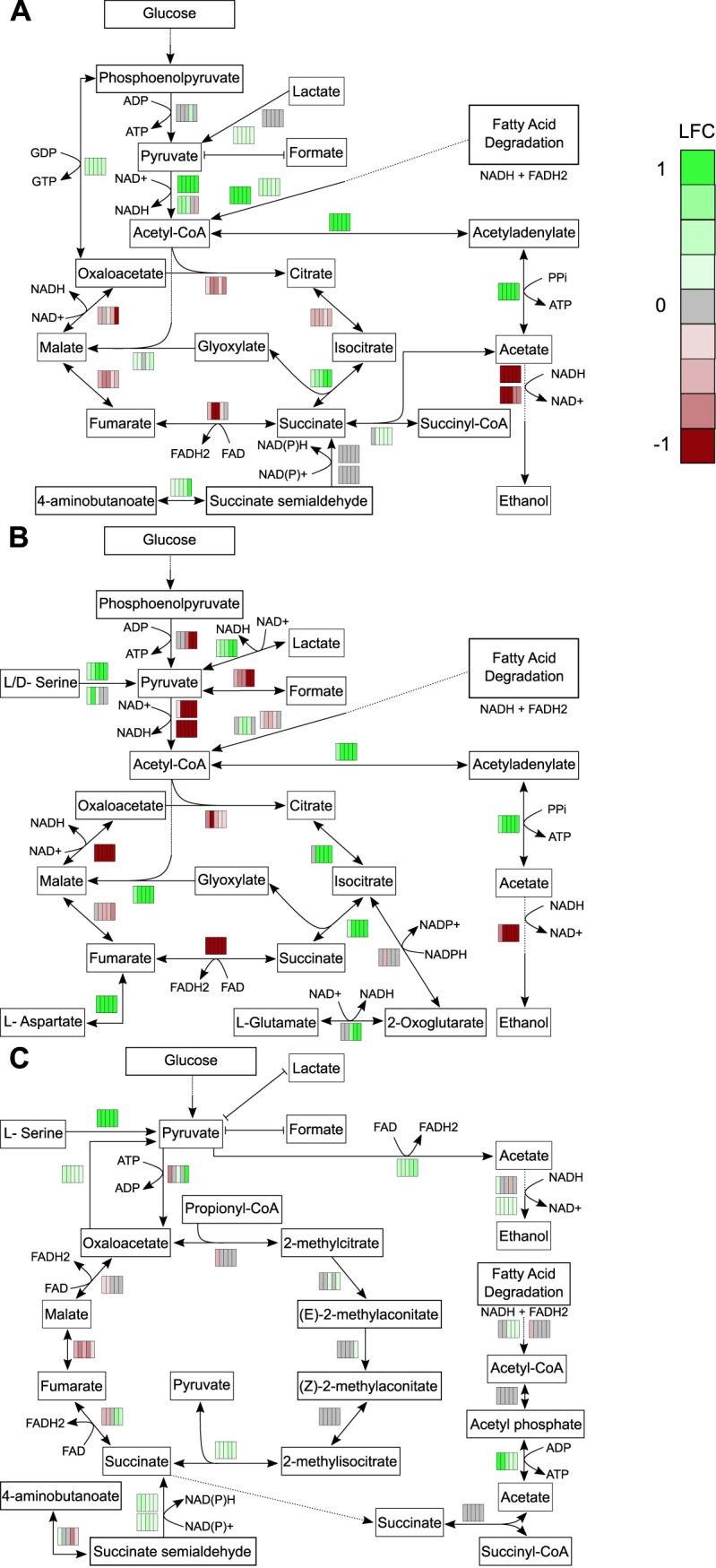
Temporal changes in transcriptomics reveal rerouting of metabolism toward succinate production. Log_2_ fold change (LFC) values were mapped onto pathways involved in succinate production for B. thailandensis (A), C. violaceum (B), and P. syringae (C). LFC values are represented by rectangles alongside each reaction in the pathway map. Each column represents the 5 stationary-phase time points. Colors within each rectangle represent LFCs (green, increased transcripts; red, decreased transcripts) compared to the exponential-phase time point. FAD, flavin adenine dinucleotide; FADH2, reduced flavin adenine dinucleotide.

Of the most accumulated exometabolites, succinate was a common exometabolite detected in all strains, and this is unsurprising as it is directly involved in central metabolism. Notably, succinate did not meet our stringent definitions of released and accumulating over stationary phase (Fig. 6). However, its abundance and accumulation for all strains and its important role in central metabolism warranted further investigation. We overlaid temporal log fold changes in gene expression onto KEGG pathways involved in succinate production (Fig. 7). These data suggest that all strains rerouted metabolism during stationary phase. For the most part, transcripts involved in glycolysis and the TCA cycle were decreased in all strains (KEGG pathways). With regard to succinate production, both B. thailandensis and C. violaceum appear to have rerouted metabolism to use the glyoxylate cycle, as supported by the increases in transcripts for isocitrate lyase and transcripts involved in the β-oxidation of fatty acids. P. syringae appears to have rerouted metabolism to use the methylcitrate cycle to generate succinate, as evidenced by the increase in transcripts for 2-methylisocitrate lyase. Other potential sources of succinate production include the γ-aminobutyric acid (GABA) shunt and succinyl-CoA:acetate-CoA transferase in both B. thailandensis and P. syringae. In all strains, stationary phase results in exometabolite production that appears to coincide with alterations in metabolism.

## DISCUSSION

Microbes can experience a feast-or-famine lifestyle in environments (e.g., soil, activated sludge, and the gut) where long periods of starvation are punctuated by short periods of nutrient flux (4–10). Thus, microbes in particular environments predominantly exist in stationary phase. Understanding the metabolic response to stationary phase can reveal generalities as well as strain-specific strategies to maintain viability in nutrient-exhausted environments.

We studied exometabolite production in stationary phase across three bacterial strains. We specifically focused our analyses on released exometabolites, metabolites that accumulated in the medium over time. Even though we applied a very conservative definition to identify features that accumulated over time, we detected and characterized thousands of features that met our criteria. However, in the end, only a subset of these features could be identified using standards, MS/MS databases, and computational predictions based on chemical characteristics (Fig. 4; see also Fig. S2 to S4 in the supplemental material and Dataset 2 at https://github.com/ShadeLab/Paper_Chodkowski_MonocultureExometabolites_2020/tree/master/Datasets).

Exometabolites could accumulate over stationary phase by two mechanisms. First, exometabolites could be transported passively or actively across viable cells’ membranes. Second, cells could lyse and spill primary metabolites and other debris into the extracellular environment (34). Our results suggest that a major factor contributing to exometabolite accumulation for all three strains investigated here was exometabolite release from intact cells. In fact, we did not observe a death phase over stationary phase (Fig. S1). Live cells generally remained at consistent levels throughout stationary phase. One generation during stationary phase was observed for both B. thailandensis and P. syringae. Given the decrease in transcripts observed for multiple genes in central metabolism (KEGG pathways), this generation was likely the result of reductive cell division (35–37). Dead cells were present and, in particular, increased for P. syringae throughout the time course. While dead cells could leak exometabolites, the accumulation of certain exometabolites (e.g., secondary metabolites) was identified and previously associated with production from viable cells in stationary-phase cultures from each strain (29, 30, 32). Furthermore, our results are consistent with a previous study in E. coli that observed the extracellular accumulation of nucleobases upon entry into stationary phase (19). Ribosome degradation is initiated in growth-limiting environments and is a likely source of nucleobase accumulation due to the degradation of rRNA (38). We also observed the accumulation of various nucleobases in the extracellular environment across all strains, consistent with the concept of some common stationary-phase phenomena among bacteria. Additional evidence of exometabolite release from intact cells was provided by transcriptome sequencing (RNA-seq) analysis. Transcriptomics results indicated an increase in transcripts for or consistent expression of transporters (see Dataset 1 at the URL mentioned above). In a previous study, Paczia et al. also observed similar patterns of exometabolite accumulation in stationary phase in various strains (39). They were able to rule out lysis and determine that passive or active diffusion could explain exometabolite production under growth-limited conditions. In integrating transcriptomics with exometabolomics, our study builds on the findings of Paczia et al. to identify transporters likely involved in exometabolite accumulation and provide insights into alterations in stationary-phase metabolism. Findings from our work and the work of Paczia et al. are in agreement with metabolic models that suggest that the extracellular accumulation of central metabolites could be attributed to costless metabolic secretions in resource-poor environments (25). Unintuitively, the release of exometabolites by viable cells, and particularly the release of central carbon intermediates, may be a common adaptation of bacteria in stationary phase. An interesting explanation is that the stoichiometry of metabolites is constrained by evolved metabolic network topology: some metabolites could be produced in excess to meet all metabolite requirements for a bacterium. Fitness trade-offs of metabolite overproduction (e.g., toxic accumulation) could be alleviated through metabolite efflux (33).

In addition to the characterization of exometabolites implicated in cooperative interactions (e.g., central carbon intermediates or quorum-sensing molecules), we also identified exometabolites implicated in competition. An antibiotic (bactobolin) with previously described bioactivity (27, 29, 40) was produced by B. thailandensis, and a siderophore/virulence factor (yersiniabactin) was produced by P. syringae (30), representing interference (direct harm to neighbors) and exploitative (indirect negative interaction) competition strategies, respectively (41, 42). These exometabolites are involved in interspecies competition but were produced in monoculture here. While we did not identify an exometabolite in C. violaceum involved in competition, we identified quorum-sensing molecules, which are linked to the production of competitive exometabolites in this strain (31, 32). Taken together, the metabolic profile of each strain was altered in stationary phase and resulted in the production of both cooperative and competitive exometabolites. The simultaneous production of both cooperative and competitive exometabolites may be an advantageous strategy to sustain kin while maintaining competition for scarce resources (25, 43). Additional studies that include coculturing experiments are needed to understand the impact that these exometabolites may have on ecological dynamics and the interplay of these biotic factors under changing environmental conditions.

Putative (MSI level 2) exometabolite identifications provided evidence for the release of dipeptides (Fig. 5B), and transcriptomics provided evidence for the differentially regulated or consistent expression of dipeptide transporters (see Dataset 1 at https://github.com/ShadeLab/Paper_Chodkowski_MonocultureExometabolites_2020/tree/master/Datasets). Hydrolysis by dipeptidyl peptidases of ribosomal proteins or the degradation of other polypeptide chains can be one source of dipeptide production. Estimates for E. coli have shown that 50 to 80% of ribosomes were degraded upon transition from exponential phase to stationary phase (38). Interestingly, another source of dipeptides may be active production. Recent studies have examined dipeptide formation by adenylation domains in nonribosomal peptide synthetases (NRPSs) (44, 45). All strains in our study have numerous NRPSs that could contribute to the production of dipeptides (see Dataset 3 at the URL mentioned above). Furthermore, one dipeptide was characterized as a cyclic dipeptide. Cyclic dipeptides can be involved in cell communication (46). Thus, the diverse chemical ecology that can be facilitated by dipeptides points to the importance of understanding how dipeptides are formed and of characterizing the environments that induce their production.

A clear limitation to our study is in the incomplete exometabolite annotations. Only 1.8% of released exometabolites could be identified. While exact molecule identifications are lagging behind the identification of new features, efforts have been put forth to chemically classify all MS/MS data (47). We used the same approach to computationally predict and classify the chemical ontology of MS/MS data not identified at MSI level 1 or level 2 (Fig. 5). Differences between *in silico* predictions of MS/MS data (MSI level 3) and MSI levels 1 and 2 were most apparent at the class level (Fig. 5A). This knowledge can be used to direct research efforts and analytical techniques to identify underrepresented classes of compounds. Targeted identification efforts for exometabolites will reveal uncharacterized biological phenomena occurring in experimental systems.

Microbes in growth-arrested states can reroute metabolism to maintain the proton motive force (PMF) and stabilize ATP levels (16). We used a combination of exometabolomics and transcriptomics to shed light on metabolic rerouting in each strain investigated. Notably, all three strains accumulated high levels of succinate, and this was further supported by RNA-seq data that showed an increase in transcripts of genes involved in succinate production (Fig. 7). We found that the major metabolic rerouting in stationary phase included transitioning to the glyoxylate cycle in B. thailandensis and C. violaceum and to the methylcitrate cycle in P. syringae. This finding, specifically for B. thailandensis, agrees with previous studies in B. thailandensis and closely related strains. Previous studies found quorum-sensing-mediated metabolic rerouting to the glyoxylate cycle during stationary phase in B. thailandensis and Burkholderia glumae as a mechanism to combat alkalinity toxicity (48, 49). Furthermore, the greatest increase in isocitrate lyase was observed in Burkholderia cenocepacia during stationary phase compared to other abiotic stressors (50). This supports the notion that rerouting metabolism to the glyoxylate cycle in stationary phase may be a shared feature among members of the genus *Burkholderia*. Prior evidence for stationary-phase metabolic rerouting in both C. violaceum and P. syringae is lacking. However, a metabolic model in C. violaceum ATCC 12472 suggested that metabolic rerouting to the glyoxylate cycle occurred in response to antibiotics in a streptomycin-resistant population (51). In support of succinate extracellular accumulation, we found that C-4 dicarboxylic acid transporters were transcriptionally active in all three strains (see Dataset 1 at https://github.com/ShadeLab/Paper_Chodkowski_MonocultureExometabolites_2020/tree/master/Datasets). It could be that succinate export is facilitated by a succinate/proton symporter for maintenance of the PMF. However, both cycles involved in succinate production do not generate ATP, and the generation of ATP is also necessary to maintain cell viability. While ATP could be generated through the production of acetate (Fig. 7), we note that we did not quantify acetate and therefore are unable to confirm this scenario. Additional studies are needed to confirm the mechanisms for maintaining cell viability during stationary phase. Regardless, combining exometabolomic and transcriptomic approaches provided increased biological interpretations that could not have been achieved by either approach in isolation. The characterization of exometabolite production and the metabolic response to stationary phase in monocultures sets the stage for understanding exometabolite-mediated interspecies interactions within a microbial community.

## MATERIALS AND METHODS

### Bacterial strains and culture conditions.

Glycerol stocks of B. thailandensis, C. violaceum, and P. syringae (Table 1) were plated on half-concentration Trypticase soy agar (TSA_50_) at 27°C for at least 24 h. Strains were inoculated in 7 ml of M9–0.2% glucose medium and grown for 16 h at 27°C at 200 rpm. Cultures were then back-diluted into 50 ml M9–0.2% glucose medium such that the exponential growth phase was achieved after 10 h of incubation at 27°C at 200 rpm. Strains were back-diluted in 50 ml M9–0.067% glucose medium to target optical densities (ODs) (B. thailandensis OD of 0.3, C. violaceum OD of 0.035, and P. syringae OD of 0.035) such that stationary phase was achieved after approximately 24 h of incubation in filter plates.

### Filter plate experiments.

We used the filter plate system to study each strain in monoculture over the course of stationary phase. Filter plate preparation was performed as previously described (28). Briefly, we used sterile filter plates with 0.22-μm-pore-size polyvinylidene difluoride (PVDF) filter bottoms (MultiScreen GV filter plate, 0.22 μm, catalog number MSGVS2210; Millipore). Prior to use, filter plates were washed three times with sterile water using a vacuum apparatus (NucleoVac 96 vacuum manifold; Clontech Laboratories). The filter of well H12 was removed with a sterile pipette tip and forceps, and 31 ml of M9–0.067% glucose medium was added to the reservoir through well H12. Each well was then filled with 130 μl of back-diluted culture in M9–0.067% glucose medium or medium only. For a given time series replicate, a custom R script (RandomArray.R [see the GitHub repository at https://github.com/ShadeLab/Paper_Chodkowski_MonocultureExometabolites_2020/tree/master/Datasets]) was used to randomize the placement of a strain in the wells so that a strain occupied a total of 31 wells per plate and the remaining 64 wells were filled with medium. Each monoculture time course was independently replicated four times for a total of 12 experiments. The time course included 6 time points: an exponential-phase point (12.5 h) and 5 points assessed every 5 h over stationary phase (25 h to 45 h). Plates were destructively sampled, comprising a total of 72 plates for the entire experimental design of 3 strains × 6 time points × 4 replicates.

Filter plates were incubated at 27°C with gentle shaking (∼0.32 relative centrifugal force [rcf]). We again used our RandomArray.R script to randomize wells used for RNA extraction (16 wells, pooled per plate) and flow cytometry (5 wells, pooled per plate). During destructive sampling, the wells containing spent culture assigned to RNA-seq were first pooled into a 1.5-ml microcentrifuge tube, flash-frozen in liquid nitrogen, and stored at −80°C for RNA extraction. Next, wells containing spent culture assigned to flow cytometry were pooled, and 20 μl was initially diluted in 180 μl Tris-buffered saline (TBS) (20 mM Tris, 0.8% NaCl [pH 7.4]) and then, after checking the concentrations needed for accurate flow cytometry counts, diluted further in TBS to reach final dilutions of 1,300-fold, 1,540-fold, and 900-fold for B. thailandensis, C. violaceum, and P. syringae, respectively. Finally, spent medium (∼31 ml) from the shared reservoir was transferred into 50-ml conical tubes, flash-frozen in liquid nitrogen, and stored at −80°C for subsequent exometabolite extraction.

### Flow cytometry.

Diluted cultures were stained with the Thermo Scientific Live/Dead BacLight bacterial viability kit at final concentrations of 1.5 μM Syto9 (live stain) and 2.5 μM propidium iodide (dead stain). Two hundred microliters of stained cultures was transferred to a 96-well microtiter U-bottom microplate (Thermo Scientific). Twenty microliters was analyzed on a BD Accuri C6 flow cytometer (BD Biosciences) at a fluidics rate of 66 μl/min and a threshold of 500 on an FL2 gate. The instrument contained the following optical filters: FL1-533, 30 nm; FL2-585, 40 nm; and FL3, 670-nm long pass. The counting accuracy of the flow cytometer was periodically checked with green fluorescent protein (GFP) beads. Data were analyzed using BD Accuri C6 software version 1.0.264.21 (BD Biosciences).

### Metabolomics. (i) LC-MS sample preparation and data acquisition.

The following methods were performed according to Department of Energy Joint Genome Institute (DOE JGI) standard operating protocols at the DOE JGI facility. Spent medium samples from the monocultures were shipped from Michigan State to the DOE JGI overnight on dry ice. Spent medium (ranging from 2.5 to 8 ml) was lyophilized in a Labconco FreeZone 2.5 lyophilizer (Labconco, Kansas City, MO). Dried samples were resuspended in 700 μl methanol, vortexed, sonicated for 10 min in a water bath (VWR Scientific Aquasonic water bath, model 150HT), and then centrifuged for 2 min at 1,200 × *g*. The supernatant was transferred to a 96-deep-well plate (1.1 ml) and then dried in a SpeedVac (catalog number SPD111V; Thermo Scientific). Samples were stored at −80°C until liquid chromatography-mass spectrometry (LC-MS) analysis. Four extraction blanks were also prepared using the same protocol.

Dried samples were resuspended in methanol containing internal standards (ITSDs). ITSD used for polar analysis was a ^13^C,^15^N amino acid mixture (30 μM) (catalog number 767964; Sigma, Inc.). The ITSD for nonpolar analysis was 2-amino-3-bromo-5-methylbenzoic acid (ABMBA) (1 μg/ml). Additionally, a quality control (QC) sample containing ∼20 common biomolecules was prepared. ITSDs are used to check for injection errors, mass accuracy, and retention time shifts within a sample. The *m/z* accuracy and retention time shifts in QC samples were assessed to check for instrument consistency and column performance. Samples were analyzed for both polar and nonpolar exometabolites. Resuspended samples containing ITSDs were vortexed, sonicated in a water bath for 2 min, transferred to transwell plates (MultiScreen GV filter plate, 0.22 μm, catalog number MSGVS2210; Millipore), centrifuged for 2 min at ∼1,200 × *g* in a 96-well plate, and then transferred into an LC-MS glass vial.

Ultrahigh-performance liquid chromatography (UHPLC) was performed using an Agilent 1290 LC stack, with MS and tandem mass spectrometry (MS2) data collected in both positive and negative ion modes using a Thermo QExactive (for hydrophilic interaction liquid chromatography [HILIC]) or a Thermo QExactive HF (for C_18_) mass spectrometer (Thermo Scientific, San Jose, CA). Full MS spectra was collected for *m/z* 80 to 1,200 at a 60,000 resolution for C_18_ and for *m/z* 70 to 1,050 at a 70,000 resolution for HILIC. MS/MS fragmentation data were acquired using stepped collision energies of between 10 and 40 eV at a 17,500 resolution. Specifically, 1 MS1 scan was followed by 2 MS2 scans of the 2 most intense ions, and another MS1 scan was then followed by another 2 MS2 scans of the 2 most intense ions. If the 2 most intense ions were already fragmented in the previous 10 s of the analysis, the next 2 most intense ions were fragmented. For MS2, 10-, 20-, and 30-eV collision energies were collected and averaged, with the exception of one biological replicate under each condition where 10-, 20-, and 40-eV collision energies were collected and averaged.

For the detection of nonpolar metabolites, reverse-phase chromatography was performed using a C_18_ column (Agilent Zorbax Eclipse Plus C_18_, Rapid Resolution HD, 2.1 by 50 mm, 1.8 μm) at a flow rate of 0.4 ml/min. Samples were run on the C_18_ column held at 60°C and equilibrated with 100% buffer A (100% LC-MS water with 0.1% formic acid) for 1 min, followed by a linear gradient to 100% buffer B (100% acetonitrile with 0.1% formic acid) over 7 min and then isocratic elution in 100% buffer B for 1.5 min. A final reequilibration to 100% buffer A over 1 min and an isocratic hold for 1 min were performed prior to the next sample injection. For the detection of polar metabolites, normal-phase chromatography was performed using a ZIC-HILIC column (SeQuant ZIC-HILIC, 3.5-μm particle size, 200-Å porosity, 150 mm by 2.1 mm; Millipore Sigma). Samples were run on the ZIC-HILIC column held at 40°C and equilibrated with 100% buffer B (95:5 acetonitrile-water with 5 mM ammonium acetate) at a flow rate of 0.45 ml/min for 1.5 min, diluting buffer B down to 65% with buffer A (100% water with 5 mM ammonium acetate) over 13.5 min, followed by a linear increase in the flow rate to 0.6 ml/min as buffer B approached 0% over 3 min and then isocratic elution in 100% buffer A for 5 min. This was followed by a 2-min linear gradient back to 100% buffer B, a decrease in the flow rate to 0.45 ml/min, and then a final 5-min column reequilibration at 100% buffer B prior to the next sample injection.

The sample injection order on the mass spectrometer was randomized, and an injection blank (2 μl of methanol) was run between each sample. For all samples, the resuspension volume (70 to 120 μl) and injection volume (2 μl to 8 μl) varied to normalize by the initial sample volume prior to extraction. A total of 257 samples were successfully analyzed (see Dataset 4 at https://github.com/ShadeLab/Paper_Chodkowski_MonocultureExometabolites_2020/tree/master/Datasets). Samples not included in downstream analyses were removed either because they failed quality standards during mass spectrometry analysis or because the sample had low intragroup reproducibility.

### (ii) Mass spectrometry analysis.

Both MS and MS/MS data were used for untargeted metabolomics analysis. A total of 257/288 metabolomic samples were used for analysis (see Dataset 4 at https://github.com/ShadeLab/Paper_Chodkowski_MonocultureExometabolites_2020/tree/master/Datasets); 30 samples were removed due to failed injection, and 1 sample was removed due to low intragroup reproducibility in polar analysis (Pearson’s *r* ≤ 0.14). MZmine (version 2.42) (52) was used for peak picking, aligning features across samples, and peak integration for both nonpolar and polar analyses and in both negative and positive ion modes. MZmine XML parameter files for all analyses can be viewed and downloaded from GitHub (see Dataset 7 at https://github.com/ShadeLab/Paper_Chodkowski_MonocultureExometabolites_2020/tree/master/Datasets). For MS data, a feature-by-sample matrix was exported for additional feature-filtering steps. For MS/MS data, the GNPS feature was used to export data in addition to performing a local spectrum database search within MZmine (see the compound identification section, below).

We used feature-filtering steps to identify exometabolites released from each strain in stationary phase. The feature-filtering steps were performed as follows on a per-strain basis: (i) features were removed if the maximum peak area was found in one of the replicates for the external control sample; (ii) a noise filter, the minimum peak area of a feature from a replicate at the last time point (45 h) needed to be 3 times the maximum peak area of the same feature in one of the external control replicates, was applied; (iii) coefficient of variation (CV) values for each feature calculated between replicates at each time point needed to be less than 20% across the time series; (iv) the minimum value of the average peak area needed to be observed in the first, exponential-phase time point (12.5 h); (v) The log_2_ fold change (LFC) of the average peak areas observed between the last (45 h) and first (12.5 h) time points needed to be greater than 1; and (vi) the time series abundance of a feature needed to have a Pearson correlation greater than or equal to 0.7.

Four final feature data sets from polar and nonpolar analyses in both ionization modes were analyzed in MetaboAnalyst 4.0 (53). Features were normalized by an ITSD reference feature (see Dataset 5 at https://github.com/ShadeLab/Paper_Chodkowski_MonocultureExometabolites_2020/tree/master/Datasets) and cube root transformed. Reference features for polar analyses in positive ([^13^C,^15^N]proline) and negative ([^13^C,^15^N]alanine) modes were determined by the ITSD with the lowest CV value across all samples. The reference feature for nonpolar data sets was the ITSD ABMBA. Heat maps were generated in MetaboAnalyst using Ward’s clustering algorithm with Euclidean distances from Z-scored data. Normalized and transformed data sets were exported from MetaboAnalyst to generate principal-coordinate analysis (PCoA) plots in R.

### (iii) Compound identification.

A three-step process was used to identify compounds or characterize chemical ontologies (47). Identification confidence was assigned according to the Metabolomics Standards Initiative (MSI) (54). First, compounds were identified by an in-house reference library at the Joint Genome Institute (JGI). This reference library was curated to identify compounds based on *m/z*, retention time, and MS/MS spectra of standards. A compound passing the first two criteria was denoted MSI level 1. A compound passing all three criteria exceeded MSI level 1. All compounds at or exceeding MSI level 1 were identified using the reference library. This reference library was available for polar analysis only. Ranges for *m/z* and retention time values for compounds in the reference library were used to identify exometabolites from the MZmine analysis (see Dataset 6 at https://github.com/ShadeLab/Paper_Chodkowski_MonocultureExometabolites_2020/tree/master/Datasets).

We made an effort to identify as many of the remaining compounds from both polar and nonpolar analyses that had MS/MS data. MS/MS data acquired during mass spectrometry analysis were used to putatively identify compounds that matched the fragmentation patterns from libraries outside the JGI; these were assigned MSI level 2. First, MS/MS data were exported to GNPS format and analyzed in GNPS (55) to match fragmentation patterns against the NIST17 commercial library. Second, a local spectrum database search was performed within MZmine using the entire compound library from the MassBank of North American (MoNA) (https://mona.fiehnlab.ucdavis.edu). For both approaches, compounds were putatively identified if cosine scores were 0.7 or above. A subset of the final feature data sets was created from compounds identified at MSI level 1 and level 2 (see Dataset 2 at https://github.com/ShadeLab/Paper_Chodkowski_MonocultureExometabolites_2020/tree/master/Datasets). These data sets were processed in MetaboAnalyst (see the mass spectrometry analysis section, above) to generate heat maps, perform pathway analysis (see the pathway analysis section, below), and perform ANOVA between the strains’ exometabolite abundances.

All remaining unidentified compounds with MS/MS data were analyzed with CSI:Finger ID and assigned MSI level 3. This method provides the putative chemical ontology of a compound. The top CSI:Finger ID match was used for each compound. Next, lnChl keys from all MSI levels were used to perform a chemical ontology analysis using ClassyFire version 1.0. SDF files from ClassyFire were exported from each analysis to extract both class-level and direct parent-level ontologies. These data were then exported to R for data visualization.

### RNA-seq. (i) RNA sample preparation, sequencing, and QC.

At Michigan State, RNA was extracted using the E.Z.N.A. bacterial RNA kit (Omega Bio-Tek, Inc.). An in-tube DNase I (2 U) (catalog number AM2222; Ambion, Inc.) digestion was performed to remove DNA from RNA samples. RNA samples were purified and concentrated using the Qiagen RNeasy MinElute cleanup kit (Qiagen, Inc.). Ten random samples were chosen to assess RNA integrity on an Agilent 2100 bioanalyzer.

The following methods were performed according to DOE JGI standard operating protocols at the DOE JGI facility. RNA samples were shipped from Michigan State to the DOE JGI overnight on dry ice. RNA samples were placed into 4, 96-well plates: 1 plate for each species containing all stationary-phase time points and 1 plate containing exponential-phase time points. Plate-based RNA sample preparation, including the Ribo-Zero rRNA removal kit (Illumina) (for bacteria) and the TruSeq stranded total RNA HT sample prep kit, was performed on the PerkinElmer Sciclone Next Generation Sequencing (NGS) robotic liquid handling system under the following conditions: total RNA starting material of 100 ng per sample and 10 cycles of PCR for library amplification. The prepared libraries were quantified using the Kapa Biosystems next-generation sequencing library quantitative PCR (qPCR) kit and run on a Roche LightCycler 480 real-time PCR instrument. The quantified libraries were then prepared for sequencing on the `Illumina HiSeq sequencing platform utilizing a TruSeq rapid paired-end cluster kit, v4. Sequencing of the flow cell was performed on the Illumina HiSeq2500 sequencer using HiSeq TruSeq SBS sequencing kits, v4, following a 2- by 100-nucleotide (nt) indexed run.

### (ii) Read preprocessing and filtering.

BBDuk (56) was used on raw fastq files to filter contaminants and trim both adaptor sequence and quality trim reads from the 3′ end of each read where quality dropped to zero. Using BBDuk, raw reads were evaluated for artifact sequences by kmer matching (kmer = 25), allowing 1 mismatch, and detected artifacts were trimmed from the 3′ ends of the reads. BBDuk was used to remove reads that contained 1 or more “N” bases, had an average quality score across the read of less than 10, or had a minimum length of ≤51 bp or 33% of the full read length. Reads that mapped with BBMap (56) to masked human, cat, dog, and mouse reference sequences at 93% identity were removed. Reads that aligned to common microbial contaminants were also removed. rRNA reads were also removed.

### (iii) Pseudoalignment and counting.

The reads from each library were pseudoaligned to the transcriptome of each strain with kallisto (57). Raw counts from each library were combined into a gene count matrix for each strain. The gene count matrix was used for downstream analyses.

### Transcriptomics. (i) RNA quality filtering and differential gene expression analysis.

Count matrices for each strain were quality filtered in two steps prior to differential gene expression (DGE) analysis: genes containing 0 counts in all samples were removed, and genes with a count of ≤10 in more than 90% of samples were removed. DGE analysis was performed in DESeq2 version 1.22.1 (58). We tested for differential gene expression by evaluating genes that changed at any time point (FDR < 0.01). Genes with differential expression were then evaluated for log_2_ fold changes of >1. Specifically, we focused on genes involved in transport (see the transporter analysis section, below).

### (ii) Defining expression minimums.

A cumulative abundance plot was generated for each strain by organizing locus identifications from low transcript counts to high transcript counts and plotting the percentage of total transcripts against the percentage of total read counts (59, 60). The 25th quantile was calculated to obtain the transcript count value that defined a low-expression minimum. That is, all genes with transcript counts above this minimum were considered to be expressed in the cell, regardless of longitudinal differential expression.

### (iii) Transporter analysis.

TransportDB 2.0 (http://www.membranetransport.org/transportDB2/index.html) was used to annotate transporters in each strain (61). Annotated transporters were then evaluated to determine differential expression or expression above the low-expression minimum.

### KEGG pathway analysis.

We extracted LFC values from transcripts in each strain from DESeq analysis. Log_2_ fold changes were obtained by comparing each stationary-phase time point to exponential-phase time point 1 (12.5 h). We then mapped longitudinal LFCs onto KEGG pathways for each strain using the pathview package in R. First, K numbers were assigned to genes for both C. violaceum and P. syringae using BlastKOALA (version 2.2). K numbers were not assigned to B. thailandensis because KEGG identifiers were available. KEGG identifiers for B. thailandensis and K numbers assigned to C. violaceum and P. syringae were used to map longitudinal LFCs onto KEGG pathways. Pathways of interest were curated and manually edited in Inkscape (version 0.92.4) using a color-blind palette.

### Annotation of biosynthetic gene clusters.

Biosynthetic gene clusters (BSGCs) were annotated using antismash bacterial version 5.0 (62). Annotated genome files for each strain were submitted to the online server. Default parameters included a relaxed detection strictness and extra features such as KnownClusterBlast, SubClusterBlast, and ActiveSiteFinder.

### Code availability.

Computing code, workflows, and data sets are available at https://github.com/ShadeLab/Paper_Chodkowski_MonocultureExometabolites_2020. R packages used during computing analyses included vegan (63), ggplot2 (64), VennDiagram (65), RVAideMemoire (66), patchwork (67), DESeq2 (58), pathview (68), KEGGREST (69), and helper functions (70–73).

### Data availability.

Genomes for B. thailandensis, C. violaceum, and P. syringae are available at the JGI Genome Portal under project IDs 1133672, 1133669, and 1133674, respectively. An improved annotated draft genome of C. violaceum is available under NCBI BioProject accession number PRJNA402426 (GenBank accession number PKBZ00000000). Data for resequencing efforts for B. thailandensis and P. syringae are under NCBI BioProject accession numbers PRJNA402425 and PRJNA402424, respectively. Metabolomics data and transcriptomics data are also available at the JGI Genome Portal (74) under JGI proposal identifier 502921. MZmine XML parameter files for all analyses can be viewed at and downloaded from GitHub (see Dataset 7 at https://github.com/ShadeLab/Paper_Chodkowski_MonocultureExometabolites_2020/tree/master/Datasets). Large data files (e.g., MZmine project files) are available upon request. Other data sets are also available on GitHub (https://github.com/ShadeLab/Paper_Chodkowski_MonocultureExometabolites_2020/tree/master/Datasets).

10.1128/mSystems.00493-20.3 FIG S3 Released and identified exometabolites and their temporal dynamics. A heat map of identified exometabolites in nonpolar positive mode is shown, where samples are in columns and exometabolites are in rows. Results for each sample are the averages from independent time point replicates (*n* = 2 to 4). Euclidean distance was calculated from Z-scored mass spectral profiles (containing peak areas). Prior to Z-scoring, features were normalized by an internal standard (ITSD) reference feature and cube root transformed. Features were clustered by Ward’s method. Download FIG S3, EPS file, 2.1 MB. Copyright © 2020 Chodkowski and Shade. 2020 Chodkowski and Shade. This content is distributed under the terms of the Creative Commons Attribution 4.0 International license.

10.1128/mSystems.00493-20.4 FIG S4 Released and identified exometabolites and their temporal dynamics. A heat map of identified exometabolites in nonpolar negative mode is shown, where samples are in columns and exometabolites are in rows. Results for each sample are the averages from independent time point replicates (*n* = 2 to 4). Euclidean distance was calculated from Z-scored mass spectral profiles (containing peak areas). Prior to Z-scoring, features were normalized by an internal standard (ITSD) reference feature and cube root transformed. Features were clustered by Ward’s method. Download FIG S4, EPS file, 1.1 MB. Copyright © 2020 Chodkowski and Shade. 2020 Chodkowski and Shade. This content is distributed under the terms of the Creative Commons Attribution 4.0 International license.
