# Supplementary material for: Exometabolite Dynamics over Stationary Phase Reveal Strain-Specific Responses
Source: mSystems. 2020 Dec 22;5(6):e00493-20. doi: 10.1128/mSystems.00493-20 (PMC7762789; doi:10.1128/mSystems.00493-20)
Supplement: TABLE S1 [file mSystems.00493-20-st001.docx]

|  | Strain | Time | Strain x Time |
| --- | --- | --- | --- |
| Polar Positive | 0.578 | 0.061 | 0.685 |
| Polar Negative | 0.670 | 0.026 | 0.749 |
| Nonpolar Positive | 0.762 | 0.024 | 0.858 |
| Nonpolar Negative | 0.800 | 0.000 | 0.865 |
