# Supplementary material for: Exometabolite Dynamics over Stationary Phase Reveal Strain-Specific Responses
Source: mSystems. 2020 Dec 22;5(6):e00493-20. doi: 10.1128/mSystems.00493-20 (PMC7762789; doi:10.1128/mSystems.00493-20)
Supplement: TABLE S2 [file mSystems.00493-20-st002.docx]

|  | m12 | R | *P* |
| --- | --- | --- | --- |
| *B. thailandensis* | 0.019 – 0.389 | 0.782 – 0.990 | 0.001 – 0.040 |
| *C. violaceum* | 0.008 – 0.190 | 0.900 – 0.996 | 0.001 – 0.035 |
| *P. syringae* | 0.016 – 0.300 | 0.837 – 0.992 | 0.001 – 0.075 |
