# Supplementary material for: Exometabolite Dynamics over Stationary Phase Reveal Strain-Specific Responses
Source: mSystems. 2020 Dec 22;5(6):e00493-20. doi: 10.1128/mSystems.00493-20 (PMC7762789; doi:10.1128/mSystems.00493-20)
Supplement: TABLE S3 [file mSystems.00493-20-st003.docx]

| Time (h) | *B. thailandensis* | *C. violaceum* | *P. syringae* |
| --- | --- | --- | --- |
| 25 | 0.132 - 0.181 | 0.232 – 0.378 | 0.233 – 0.374 |
| 30 | 0.148 - 0.215 | 0.298 – 0.382 | 0.303 – 0.436 |
| 35 | 0.179 - 0.265 | 0.326 – 0.442 | 0.339 – 0.458 |
| 40 | 0.218 - 0.323 | 0.381 – 0.521 | 0.370 – 0.506 |
| 45 | 0.242 - 0.333 | 0.361 – 0.526 | 0.391 – 0.519 |
