# Supplementary material for: Exometabolite Dynamics over Stationary Phase Reveal Strain-Specific Responses
Source: mSystems. 2020 Dec 22;5(6):e00493-20. doi: 10.1128/mSystems.00493-20 (PMC7762789; doi:10.1128/mSystems.00493-20)
Supplement: TABLE S4 [file mSystems.00493-20-st004.docx]

| Time comparisons (h) | *B. thailandensis* | *C. violaceum* | *P. syringae* |
| --- | --- | --- | --- |
| 25 to 12.5 | 0.132 - 0.181 | 0.232 – 0.378 | 0.233 – 0.374 |
| 30 to 25 | 0.036 – 0.056 | 0.035 – 0.112 | 0.070 – 0.096 |
| 35 to 30 | 0.041 – 0.064 | 0.042 – 0.078 | 0.032 – 0.058 |
| 40 to 35 | 0.029- 0.083 | 0.066 – 0.097 | 0.049 – 0.075 |
| 45 to 40 | 0.023 - 0.052 | 0.023 – 0.052 | 0.036 – 0.057 |
