# Supplementary material for: Exometabolite Dynamics over Stationary Phase Reveal Strain-Specific Responses
Source: mSystems. 2020 Dec 22;5(6):e00493-20. doi: 10.1128/mSystems.00493-20 (PMC7762789; doi:10.1128/mSystems.00493-20)
Supplement: TABLE S5 [file mSystems.00493-20-st005.docx]

|  | *B. thailandensis* | *C. violaceum* | *P. syringae* |
| --- | --- | --- | --- |
| Polar Positive | 0.001 (0.553) | 0.001 (0.644) | 0.001 (0.626) |
| Polar Negative | 0.068 (0.363) | 0.001 (0.650) | 0.002 (0.630) |
| Nonpolar Positive | 0.003 (0.744) | 0.002 (0.746) | 0.001 (0.892) |
| Nonpolar Negative | 0.001 (0.849) | 0.001 (0.877) | 0.001 (0.893) |
