# Supplementary material for: Exometabolite Dynamics over Stationary Phase Reveal Strain-Specific Responses
Source: mSystems. 2020 Dec 22;5(6):e00493-20. doi: 10.1128/mSystems.00493-20 (PMC7762789; doi:10.1128/mSystems.00493-20)
Supplement: TABLE S6 [file mSystems.00493-20-st006.docx]

| Time comparisons (h) | *B. thailandensis* | *C. violaceum* | *P. syringae* |
| --- | --- | --- | --- |
| 25 to 12.5 | 0.093 – 0.21 | 0.088 – 0.15 | 0.075 – 0.17 |
| 30 to 12.5 | 0.093 – 0.21 | 0.088 – 0.13 | 0.075 – 0.17 |
| 35 to 12.5 | 0.093 – 0.21 | 0.088 – 0.13 | 0.075 – 0.17 |
| 40 to 12.5 | 0.093 – 0.21 | 0.088 – 0.15 | 0.075 – 0.17 |
| 45 to 12.5 | 0.093 – 0.21 | 0.088 – 0.15 | 0.075 – 0.17 |
| 30 to 25 | 0.75 – 0.89 | 0.38 – 1.0 | 0.15 – 0.79 |
| 35 to 25 | 0.15 – 0.86 | 0.16 – 0.28 | 0.15 – 0.72 |
| 40 to 25 | 0.098 – 0.69 | 0.088 – 0.2 | 0.075 – 0.59 |
| 45 to 25 | 0.12 – 0.69 | 0.088 – 0.2 | 0.086 – 0.40 |
| 35 to 30 | 0.75 – 0.89 | 0.49 – 0.98 | 0.64 – 1.0 |
| 40 to 30 | 0.15 – 0.75 | 0.13 – 0.45 | 0.15 – 0.79 |
| 45 to 30 | 0.15 – 0.75 | 0.11 – 0.59 | 0.15 – 0.60 |
| 40 to 35 | 0.38 – 0.96 | 0.16 – 0.56 | 0.46 – 0.97 |
| 45 to 35 | 0.27 – 0.96 | 0.15 – 0.88 | 0.38 – 0.83 |
| 45 to 40 | 0.90 – 0.96 | 0.87 – 1.0 | 0.94 – 1.0 |
